# Cefiderocol Resistance in Klebsiella pneumoniae Is Linked to SHV Extended-Spectrum β-Lactamase Activities and Functional Loss of the Outer Membrane Porin OmpK35

**DOI:** 10.1128/spectrum.03496-22

**Published:** 2023-04-25

**Authors:** Sun Hee Moon, En Huang

**Affiliations:** a Department of Environmental Health Sciences, University of Arkansas for Medical Sciences, Little Rock, Arkansas, USA; Johns Hopkins University School of Medicine

**Keywords:** cefiderocol, resistance, *bla*_SHV_, avibactam, *Klebsiella pneumoniae*

## Abstract

Klebsiella pneumoniae AR 0047 from the CDC and FDA Antibiotic Resistance Isolate Bank is resistant to cefiderocol, a siderophore-conjugated cephalosporin. Genomics analysis and genetic complementation revealed that a frameshift mutation in *ompK35* contributed to cefiderocol resistance. Heterologous expression of *bla*_SHV-5_ or *bla*_SHV-12_ in Escherichia coli increased the host resistance to cefiderocol. Moreover, avibactam, a β-lactamase inhibitor, enhanced cefiderocol activity against the resistant strain. Therefore, cefiderocol resistance is linked to SHV and the loss of *ompK35*.

**IMPORTANCE** Understanding cefiderocol resistance mechanisms is essential for providing solutions to treat infections and to prevent resistance development. Cefiderocol resistance in Klebsiella pneumoniae AR 0047 is linked to SHV β-lactamase activities and functional loss of outer membrane porin. The cefiderocol-avibactam combination represents an opportunity to increase potency against cefiderocol-resistant pathogens.

## INTRODUCTION

Extended-spectrum β-lactamase-producing and carbapenem-resistant members of *Enterobacterales* are recognized as a threat to public health (1). There is an urgent need to develop and use new effective antimicrobial agents for the treatment of infections caused by multidrug-resistant pathogens. Cefiderocol, which received FDA approval in 2019, is a novel siderophore-conjugated cephalosporin with activity against carbapenem-resistant pathogens (2, 3). The new antibiotic is indicated for the treatment of complicated urinary tract infections, hospital-acquired bacterial pneumonia, and ventilator-associated bacterial pneumonia. Like other β-lactam antibiotics, cefiderocol binds to penicillin-binding proteins (PBPs), inhibiting peptidoglycan synthesis. The siderophore molecule uses the active iron uptake system for cell entry (4). In addition to its unique mode of cell entry, cefiderocol showed excellent activity in the presence of a variety of β-lactamases (5, 6). Cefiderocol-nonsusceptible isolates were reported in surveillance studies, but the resistance mechanisms were not thoroughly studied (5, 6). Reduced susceptibility could be associated with β-lactamases and other unidentified factors (7). This objective of this study was to investigate the mechanisms of cefiderocol resistance in a carbapenem-resistant isolate, Klebsiella pneumoniae AR 0047, from the CDC and FDA Antibiotic Resistance Isolate Bank.

## RESULTS AND DISCUSSION

### Resistance genes in K. pneumoniae AR 0047.

Cefiderocol-resistant K. pneumoniae AR 0047 was identified when testing cefiderocol susceptibility using the disk diffusion method against the isolates in the Gram-negative carbapenemase detection panel from the CDC and FDA Antibiotic Resistance Isolate Bank (8). K. pneumoniae AR 0047 belongs to sequence type 258 (ST258), as determined by multilocus sequence typing using MLST 2.0 (https://cge.food.dtu.dk/services/MLST/). Antimicrobial resistance genes in K. pneumoniae AR 0047 were identified using the Resistance Gene Identifier (RGI). The resistance genes included *bla*_SHV-5_, *bla*_SHV-12_, *bla*_SHV-11_, *bla*_TEM-1_, *aac(6′)-Ib10*, *tet*(D), *mphA*, *sul1*, *aadA2*, *dfrA12*, *catI*, *fosA6*, and several antibiotic efflux genes. Genomics analysis revealed that no deletions or early terminations were found in the well-characterized siderophore uptake genes (such as *cirA*, *tonB*, *fiu*, *fhuA*, *exbB*, *exbD*, *fepA*, *fepB*, *fepC*, and *fepD*) in K. pneumoniae AR 0047 compared to the reference strain K. pneumoniae ATCC 43816 (9). However, K. pneumoniae AR 0047 possesses a single nucleotide insertion in *ompK35* at position 121, resulting in a premature stop codon (TGA) and truncation in OmpK35. It has been reported that OmpK35 truncation occurred ubiquitously in K. pneumoniae ST258 (10, 11). We hypothesized that the presence of SHV extended-spectrum β-lactamases, along with the functional loss of OmpK35, may contribute to cefiderocol resistance in K. pneumoniae AR 0047.

### Loss of the outer membrane porin OmpK35 contributes to cefiderocol resistance.

To determine the impact of porin loss, the functional porin gene *ompK35* was amplified from the reference strain K. pneumoniae ATCC 13883 and cloned into pCR-BluntII-TOPO, followed by introduction into K. pneumoniae AR 0047 by electroporation. After selection on Zeocin plates, we determined the cefiderocol susceptibility of selected complemented isolates with the functional porin gene *ompK35* using the MIC test. The MIC of K. pneumoniae AR 0047 with *ompK35* truncation showed an MIC of >32 μg/mL. Genetic complementation by the functional *ompK35* gene increased the cefiderocol susceptibility. In two derivative strains complemented with functional *ompK35*, the MIC decreased to 4 to 8 μg/mL (Table 1). Therefore, OmpK35 porin loss contributed to cefiderocol resistance in K. pneumoniae AR 0047.

**TABLE 1:** MICs of cefiderocol

Species	Strain/plasmid	β-lactamase(s) and porin	MIC (μg/mL)
K. pneumoniae	AR 0047	SHV-5, SHV-12, SHV-11, TEM-1, and truncated *ompK35*	>32
K. pneumoniae	AR 0047/pCR*ompK35*_1	SHV-5, SHV-12, SHV-11, TEM-1, and functional *ompK35* by complementation	4–8
K. pneumoniae	AR 0047/pCR*ompK35*_6	SHV-5, SHV-12, SHV-11, TEM-1, and functional *ompK35* by complementation	4–8
E. coli	TOP10/pCR*bla*_SHV-5_	SHV-5	4–8
E. coli	TOP10/pCR*bla*_SHV-12_	SHV-12	8
E. coli	TOP10/pCR*bla*_SHV-11_	SHV-11	0.5–1
E. coli	TOP10/pC*bla*_TEM-1_	TEM-1	<0.125
E. coli	TOP10/pCR*bla*_SHV-1_	SHV-1	0.5–1
E. coli	TOP10		<0.125
E. coli	ATCC 25922		0.125–0.25

### SHV extended-spectrum β-lactamases contribute to cefiderocol resistance.

Four β-lactamase genes (*bla*_SHV-5_, *bla*_SHV-12_, *bla*_SHV-11_, and *bla*_TEM-1_) were identified in the genome of K. pneumoniae AR 0047. However, the contribution of individual β-lactamases to cefiderocol resistance is unknown. Multidrug resistance to commonly used antibiotic selection markers in K. pneumoniae AR 0047 makes it difficult to inactivate multiple β-lactamase genes for functional analysis. Alternatively, we compared the impact of individual β-lactamase genes on cefiderocol susceptibility by heterologous expression in Escherichia coli. E. coli cells carrying the extended-spectrum β-lactamases *bla*_SHV-5_ or *bla*_SHV-12_ showed an increase in cefiderocol MIC compared to the control strain E. coli TOP10 (Table 1). Compared to SHV-1, SHV-5 and SHV-12 with G238S and E240K substitutions (see Table S1 in the supplemental material) contributed to a higher resistance to cefiderocol. The same amino acid substitutions are involved in resistance to other β-lactam antibiotics (12). These results suggest that the combination of two extended-spectrum β-lactamases (*bla*_SHV-5_ and *bla*_SHV-12_) may contribute to the baseline cefiderocol nonsusceptibility in K. pneumoniae AR 0047.

### Avibactam enhances the activity of cefiderocol.

**FIG 1:**
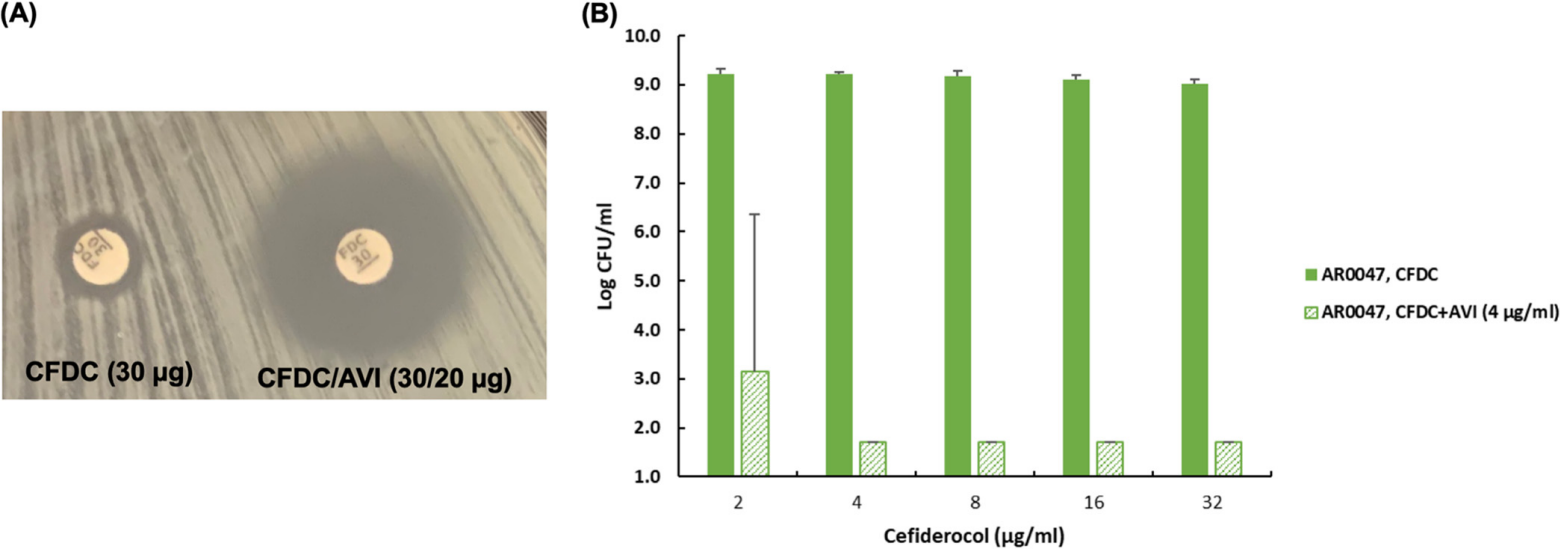
Avibactam, a β-lactamase inhibitor, enhanced the activity of cefiderocol against the cefiderocol-resistant isolate K. pneumoniae AR 0047. (A) Double-disk diffusion assay of cefiderocol (30 μg) alone (left) or with avibactam (20 μg) (right). (B) Bactericidal activity *in vitro* at 24 h. CFDC, cefiderocol alone (2 to 32 μg/mL); CFDC+AVI, cefiderocol (2 to 32 μg/mL) with avibactam at a fixed concentration (4 μg/mL).

Using the double-disk synergistic assay, we found that the β-lactamase inhibitor avibactam restored the activity of cefiderocol against cefiderocol-resistant K. pneumoniae AR 0047 (Fig. 1A). To further confirm the impact of avibactam on cefiderocol susceptibility in K. pneumoniae AR 0047, cefiderocol bactericidal activity *in vitro* was determined with or without avibactam at a fixed concentration of 4 μg/mL. When used alone, cefiderocol did not effectively inhibit the growth of K. pneumoniae AR 0047 cells. In the presence of avibactam at 4 μg/mL, the MIC of cefiderocol was 1 to 4 μg/mL. The combination of cefiderocol (≥4 μg/mL) and avibactam (4 μg/mL) eradicated almost all bacterial cells (Fig. 1B). The results suggest that β-lactamases contribute to cefiderocol resistance in K. pneumoniae AR 0047.

In summary, cefiderocol resistance in K. pneumoniae AR 0047 is linked to SHV extended-spectrum β-lactamase activities and the functional loss of the outer membrane porin OmpK35. Bacterial cells carrying *bla*_SHV-5_ and/or *bla*_SHV-12_ may develop cefiderocol resistance when mutations that reduce cefiderocol uptake occur after prolonged antibiotic exposure. Moreover, from the application perspective, the cefiderocol-avibactam combination represents an excellent opportunity to increase potency against cefiderocol-resistant pathogens.

There are some limitations in the study. We acknowledge that the plasmid-based complementation used in this study may have resulted in overexpression of the *ompK35* gene in the complemented cells. Heterologous expression of β-lactamase genes in E. coli informed the impact of each β-lactamase gene on cefiderocol resistance, even though the expression levels in E. coli may differ from those in the clinical isolate. Nonetheless, this approach can yield valuable insights into the relative activities of each β-lactamase gene within the same resistant host.

## MATERIALS AND METHODS

### Bacterial strains.

The Gram-negative carbapenemase detection panel, including K. pneumoniae AR 0047, was obtained from the CDC and FDA Antibiotic Resistance Isolate Bank (13). The genome of K. pneumoniae AR 0047 was sequenced by the strain provider and is available under GenBank accession number GCF_002180085.1. One Shot competent TOP10 E. coli cells for cloning were purchased from Invitrogen.

### Cefiderocol susceptibility tests.

Cefiderocol susceptibility was determined using the standard CLSI disk diffusion method (14) with a cefiderocol disk (30 μg; Hardy Diagnostics). The MIC of cefiderocol was determined using the CLSI broth microdilution method (14). Cefiderocol uses the active iron uptake pathway to enter cells, and iron transporters are upregulated in bacteria in iron-depleted conditions. Iron-depleted cation-adjusted Mueller-Hinton broth (ID-CAMHB) was prepared using Chelex 100 resin (Bio-Rad), which removed cations from the CAMHB medium. The iron-depleted broth was then supplemented with CaCl_2_, MgCl_2_, and ZnSO_4_ to final concentrations of 20 μg/mL, 10 μg/mL, and 0.56 μg/mL, respectively (14).

### Complementation of the outer membrane porin gene *ompK35* in K. pneumoniae AR 0047.

A point mutation in the outer membrane porin gene *ompK35* in the cefiderocol-resistant strain K. pneumoniae AR 0047 was identified using the BLSTN program for sequence alignment with the same porin gene in the reference strain K. pneumoniae ATCC 43816. To confirm the loss of function of *ompK35* in cefiderocol resistance in K. pneumoniae AR 0047, the wild-type *ompK35* gene from K. pneumoniae ATCC 13883 was cloned and used for the genetic complementation assay. Briefly, the coding region of *ompK35* from K. pneumoniae ATCC 13883 was amplified using Platinum SuperFi II DNA polymerase (Invitrogen). The PCR products were cloned into the pCR-Blunt II-TOPO vector, followed by transformation into chemically competent One Shot TOP10 E. coli cells. The plasmid carrying the wild-type *ompK35* was then transferred into K. pneumoniae AR 0047 by electroporation, followed by selection at 50 μg/mL Zeocin. The cefiderocol MICs of confirmed transformants carrying the wild-type *ompK35* gene were determined and compared to the MIC of K. pneumoniae AR 0047 without complementation.

### Cloning of β-lactamase genes into E. coli cells.

Antimicrobial resistance genes in K. pneumoniae AR 0047 were identified using the Resistance Gene Identifier (RGI) (https://card.mcmaster.ca/analyze/rgi). There are four β-lactamase genes (*bla*_SHV-5_, *bla*_SHV-12_, *bla*_SHV-11_, and *bla*_TEM-1_) in the genome of K. pneumoniae AR 0047. The coding region of *bla*_SHV-5_ was amplified from K. pneumoniae AR 0047 using Platinum SuperFi II DNA polymerase and cloned into a pCR-Blunt II-TOPO vector, followed by transformation into chemically competent One Shot TOP10 E. coli cells. The transformants were selected on kanamycin plates. The other three β-lactamase genes, *bla*_SHV-12_ from K. pneumoniae AR 0043 and *bla*_SHV-11_ and *bla*_TEM-1_ from K. pneumoniae AR 0097 (8), which encode the same β-lactamases in K. pneumoniae AR 0047, were previously cloned into E. coli cells. Another β-lactamase gene, *bla*_SHV-1_ from K. pneumoniae AR 0042, was also cloned into E. coli cells for comparison. The cefiderocol MICs of the transformants were determined and compared to that of the control TOP10 E. coli strain without transformation.

### Determination of synergistic effect with avibactam by double-disk assay.

In conjunction with the standard disk diffusion susceptibility test, we developed a simple double-disk assay to identify agents that work synergistically with cefiderocol against cefiderocol-nonsusceptible isolates. The method can rapidly identify the involvement of β-lactamases in cefiderocol resistance. Briefly, a cefiderocol disk (30 μg) was supplemented with the β-lactamase inhibitor avibactam (20 μg). Then, a cefiderocol disk and a cefiderocol-avibactam disk were plated onto Mueller-Hinton agar inoculated with the test strain, followed by incubation at 35°C for 18 h (Fig. 1A). An increase in the diameter of the inhibition zone of the cefiderocol-avibactam disk suggested that there is a synergistic effect between cefiderocol and avibactam against the test strain.

### Bactericidal activity of cefiderocol in the presence of avibactam.

The bactericidal activity of cefiderocol against K. pneumoniae AR 0047 was determined in the presence of avibactam at a fixed concentration of 4 μg/mL. Cefiderocol was used at concentrations from 2 to 32 μg/mL. After 24 h of incubation at 35°C, the bacterial populations were enumerated.

### Data availability.

The genome of K. pneumoniae AR 0047 is available publicly under accession number GCF_002180085.1.
