# Supplementary material for: Cefiderocol Resistance in Klebsiella pneumoniae Is Linked to SHV Extended-Spectrum β-Lactamase Activities and Functional Loss of the Outer Membrane Porin OmpK35
Source: Microbiol Spectr. 2023 Apr 25;11(3):e03496-22. doi: 10.1128/spectrum.03496-22 (PMC10269512; doi:10.1128/spectrum.03496-22)
Supplement: Supplemental file 1 — Supplemental material. Download spectrum.03496-22-s0001.pdf, PDF file, 0.05 MB [file spectrum.03496-22-s0001.pdf]

Table S1. Comparison of various SHV β-lactamases

	Amino acid residue at position 35	Amino acid residue at positions 238 and 240
SHV-1	L	GE
SHV-11	**Q**	GE
SHV-5	L	**SK**
SHV-12	**Q**	**SK**
